# Stakeholder-led understanding of the implementation of digital technologies within heart disease diagnosis: a qualitative study protocol

**DOI:** 10.1136/bmjopen-2023-072952

**Published:** 2023-06-27

**Authors:** Kamilla Abdullayev, Timothy JA Chico, Matthew Manktelow, Oliver Buckley, Joan Condell, Richard J Van Arkel, Vanessa Diaz, Faith Matcham

**Affiliations:** 1 School of Psychology, University of Sussex, Brighton, UK; 2 Department of Infection, Immunity and Cardiovascular Disease, The Medical School, The University of Sheffield, Sheffield, UK; 3 School of Computing, Engineering and Intelligent Systems, University of Ulster at Magee, Londonderry, UK; 4 School of Computing Sciences, University of East Anglia, Norwich, UK; 5 Department of Mechanical Engineering, Imperial College London, London, UK; 6 Department of Mechanical Engineering, University College London, London, UK; 7 Wellcome/EPSRC Centre for Interventional and Surgical Sciences, University College London, London, UK

**Keywords:** Adult cardiology, MENTAL HEALTH, PREVENTIVE MEDICINE

## Abstract

**Introduction:**

Cardiovascular diseases are highly prevalent among the UK population, and the quality of care is being reduced due to accessibility and resource issues. Increased implementation of digital technologies into the cardiovascular care pathway has enormous potential to lighten the load on the National Health Service (NHS), however, it is not possible to adopt this shift without embedding the perspectives of service users and clinicians.

**Methods and analysis:**

A series of qualitative studies will be carried out with the aim of developing a stakeholder-led perspective on the implementation of digital technologies to improve holistic diagnosis of heart disease. This will be a decentralised study with all data collection being carried out online with a nationwide cohort. Four focus groups, each with 5–6 participants, will be carried out with people with lived experience of heart disease, and 10 one-to-one interviews will be carried out with clinicians with experience of diagnosing heart diseases. The data will be analysed using an inductive thematic analysis approach.

**Ethics and dissemination:**

This study received ethical approval from the Sciences and Technology Cross Research Council at the University of Sussex (reference ER/FM409/1). Participants will be required to provide informed consent via a Qualtrics survey before being accepted into the online interview or focus group. The findings will be disseminated through conference presentations, peer-reviewed publications and to the study participants.

**STRENGTHS AND LIMITATIONS OF THIS STUDY**

- The study materials have been informed by patient advisory boards, meaning they are sensitive to the experiences of the participants and the clinicians that will be recruited.
- The study will allow an in-depth understanding of the attitudes and experience of people with lived experience of heart disease and clinicians with experience of diagnosing heart disease.
- The use of an online research platform for participant recruitment will disadvantage certain demographic and clinical groups who are less comfortable using online resources.
- The use of thematic analysis will not be free from the influence of the researcher’s personal experience and knowledge.

## Introduction

Cardiovascular diseases (CVDs) are the leading cause of global deaths and are highly prevalent across the world.^1^ The situation in the UK is no different, with the British Heart Foundation reporting 7.6 million people living in the UK with a heart and circulatory disease in August 2022, and approximately 460 deaths a day.^2^ In addition to debilitating cardiovascular symptoms, individuals suffering from CVDs often experience a diminished quality of life,^3–5^ financial burdens from medication and treatments,^6–9^ and both physical^10–12^ and psychological^13–16^ comorbidities.

Although CVDs are highly prevalent and potentially very damaging, they can become considerably more manageable, or even preventable, if diagnosed early enough.^17^ Therefore, implementing secondary prevention measures, in the form of early diagnosis and interventions, is the most effective way of reducing the life-threatening or long-term impacts of CVDs on patient health and well-being.^18^ Technological advancements within artificial intelligence and data mining may provide the tools for more accurate and effective diagnosis,^19 20^ and can contribute to reducing cardiovascular mortality.^21 22^

In addition to facilitating earlier and more accurate detection of CVDs through artificial intelligence, the increased use of ‘wearable’ ambulatory assessment technology within healthcare has provided solutions for groups facing barriers that are preventing access to primary care, such as transportation limitations in remote locations,^23 24^ or reduced mobility or capacity due to mental illness.^25–27^ As a result, those who would have missed the opportunity to receive a timely diagnosis and appropriate treatment, are no longer being excluded due to logistical limitations. Overall, it is clear that the use of remote monitoring technology in heart disease can increase the likelihood of survival and decrease the burden of CVD on the individual and ultimately on healthcare services.^28 29^

A variety of digital tools that allow more remote healthcare are already integrated into the NHS. For example, patient records can be accessed remotely thanks to use electronic databases; patients’ heart rhythms can be monitored over 24 hours from their homes using Holter monitors; and many appointments are carried out on videoconferencing software or telephone calls. Although there is common complaint regarding the quality and modernity of technology being used within the NHS, it is evident—particularly since the COVID-19 pandemic^30 31^—that there has been a rapid increase in the adoption of digital technology within both the NHS^32^ and across the world.^33^ Accordingly, there has also been increased research activity investigating the use of digital technologies in cardiovascular health.^34^

When examining the growing use of digital health technologies, it is important to highlight the distinction between the types of technologies that are being implemented, specifically between medical grade devices and consumer technologies. Medical-grade devices require a specific regulatory approval to be used and can come in many forms, including implanted defibrillators and pacemakers, and 24-hour ECG. The data collected by these devices is usually physiological and requires specialists to monitor and interpret. On the other hand, consumer technologies can include health apps on our smartphones, which track our sleep, step-count and medication adherence. These types of technologies are usually in the control of the individual, allowing self-monitoring of symptoms and self-management of many conditions, meaning there is less dependence on an already-overwhelmed healthcare system. Therefore, when the increased accuracy of data produced by medical-grade devices is combined with the detailed and personalised information collected by lifestyle technologies, it allows for a more holistic account of patient health to facilitate an accurate and efficient diagnosis of potential heart diseases.

Nevertheless, along with the potential for improving the accuracy and efficiency of diagnosis, the implementation of digital technologies into healthcare poses challenges with usability, feasibility and accessibility for patients,^35–37^ particularly those from under-represented groups in society.^38 39^ Too often, devices and systems are created without enough information regarding the patients’ and clinicians’ needs, which ultimately results in poor engagement and low cost-effectiveness.^35^ This highlights the importance of using the voices of stakeholders to inform the development and dissemination of digital health tools into cardiovascular care, as well as any other field within healthcare, otherwise the likelihood of positive effects is very low.

Therefore, a key factor in the growing shift towards the use of digital health tools within any care pathway is to gain a deeper understanding into how the new technology will fit into the existing systems, and to consider the barriers or facilitators to positive engagement from both patients and clinicians.

### Study objectives

The aim of this project is to develop a stakeholder-led understanding of the implementation of digital technologies to improve holistic diagnosis of heart disease. We have set the following three objectives which will be met via a series of qualitative studies with clinicians and individuals with lived experience of heart disease:

1. Develop a deeper understanding of stakeholder experiences of heart disease diagnosis.
2. Understand stakeholder perspectives on the use of digital health tools within the heart disease diagnosis process.
3. Explore the most meaningful and useful way of relaying digital data back to patients and clinicians.

## Methods and analyses

### Patient and public involvement

Prior to the starting participant recruitment, this research was reviewed by a cardiovascular patient advisory group based in Sheffield, which involved all participant-facing documents, including the recruitment materials and focus group schedules. This means we can be confident that the language we will use in the focus groups will be accessible and easy to understand, as well as ensuring that we are covering the important aspects of patient experience during diagnosis and when being asked to use digital health tools by their healthcare providers.

We also met with the NIHR Maudsley Biomedical Research Centre’s Race, Ethnicity and Diversity advisory group to discuss the implementation of digital technologies into heart disease diagnosis from a cultural and ethnicity perspective. This meeting highlighted the importance of considering how cultural and religious attitudes would affect engagement with digital technologies aiming to collect health data, as well as emphasising the role of family in individuals’ healthcare among ethnic minorities. Given the potential exclusion of certain populations because of our recruitment method, this insight will provide a deeper understanding of additional factors that might contribute to the acceptance and engagement of digital technologies aimed to improve holistic diagnosis of heart disease.

### Study design

This will be a qualitative study, which will use both focus group and interview designs. The topic guides were developed based on the study objectives, with an even split between (1) patient/clinician experiences of diagnosis and (2) perspectives on the use of digital technologies within healthcare.

### Study population

Based on the available time for data collection against the wider project deadlines, we plan to conduct four focus groups with individuals with lived experience of heart disease. We aim to recruit 5–6 individuals per focus group to ensure there is enough time for each participant to share their views and experiences. The following inclusion/exclusion criteria will be applied: Inclusion criteria: Lifetime diagnosis of heart disease (including but not limited to: angina, heart failure, valve disease, abnormal heart rhythms); aged 18 or over; able to speak English at a level sufficient for participation; able to give informed consent for participation. Exclusion criteria: Major cognitive impairment or dementia preventing participation.

Based on the research team’s previous experience of conducting qualitative research with clinicians, a pragmatic decision has been made to aim for a target of 10 clinicians who have had any experience (past or present) of diagnosing people with heart disease to be enrolled into interviews. The eligibility criteria for inclusion are at least 6 months of experience in working with heart disease patients in any capacity (including cardiology, nursing, primary care or other healthcare professional in multidisciplinary teams); aged 18 or over; able to speak fluent English; and able to give informed consent for participation.

### Procedure

As we are interested in hearing from participants across a range of disease durations (ie, those with very early symptoms and those who are on established management plans), we plan to recruit via: social media platforms such as LinkedIn, Twitter and Instagram; Prolific; and existing cohorts of people from the investigators’ previous research studies who have consented to be contacted for research purposes. Clinicians will be recruited using purposive sampling via personal and professional connections, and social media platforms such as LinkedIn, Twitter and Instagram.

All focus groups and interviews will be carried out online over Zoom, and consent and baseline demographic data will also be collected via online Qualtrics surveys prior to the online study. We expect focus groups to take about 2 hours and interviews to take about 1 hour. The focus groups and interviews will be semistructured and will follow a preapproved question schedule, split into two sections—patient experience of heart disease and views on digital technologies within healthcare (see online supplemental appendix 1). The same researcher will facilitate all the sessions.

10.1136/bmjopen-2023-072952.supp1 Supplementary data

Recruitment and data collection began after ethical approval on 14 November 2022 and will continue until the end of March 2023.

### Data analysis plan

Descriptive statistics for demographics, current mental distress levels, confidence using technologies and participant type-specific questions (including length of time in clinical role for clinicians and details on health condition for participants with lived-experience of heart disease) will be presented.

Transcriptions from both focus group and interview recordings will be validated by the team of researchers and coded and analysed using the NVivo software. In line with Braun and Clarke’s 2006 recommendations,^40^ an inductive thematic analysis approach will be taken, whereby the data from the transcripts will decide the themes, instead of basing them on any previous theoretical basis.

### Ethics and dissemination

This study was reviewed and approved by the Sciences & Technology Cross-School Research Ethics Council at the University of Sussex (reference ER/FM409/1) on 14 November 2022. We intend to write the resulting paper according to the Consolidated Criteria for Reporting Qualitative research guidelines.^41^

## Supplementary Material

Reviewer comments

Author's manuscript

## References

[1] World Health Organisation . Cardiovascular diseases (Cvds) [Internet]. 2011. Available: https://www.who.int/news-room/fact-sheets/detail/cardiovascular-diseases-(cvds)

[2] Health Intelligence Team B . Our vision is a world free from the fear of heart and circulatory diseases; 2022. UK Factsheet

[3] Stafford M , Soljak M , Pledge V , et al . Socio-economic differences in the health-related quality of life impact of cardiovascular conditions. Eur J Public Health 2012;22:301–5. 10.1093/eurpub/ckr007 21398378 PMC3358629

[4] Pandya A , Gaziano TA , Weinstein MC , et al . More Americans living longer with cardiovascular disease will increase costs while lowering quality of life. Health Aff 2013;32:1706–14. 10.1377/hlthaff.2013.0449 PMC3894660 24101059

[5] Tušek-Bunc K , Petek D . Comorbidities and characteristics of coronary heart disease patients: their impact on health-related quality of life. Health Qual Life Outcomes 2016;14:159. 10.1186/s12955-016-0560-1 27846850 PMC5111348

[6] Annapureddy A , Valero-Elizondo J , Khera R , et al . Association between financial burden, quality of life, and mental health among those with atherosclerotic cardiovascular disease in the United States. Circ Cardiovasc Qual Outcomes 2018;11. 10.1161/CIRCOUTCOMES.118.005180 30571331

[7] Li C , Young BR , Jian W . Association of socioeconomic status with financial burden of disease among elderly patients with cardiovascular disease: evidence from the China health and retirement longitudinal survey. BMJ Open 2018;8:e018703. 10.1136/bmjopen-2017-018703 PMC5875679 29567841

[8] Huffman MD , Rao KD , Pichon-Riviere A , et al . A cross-sectional study of the microeconomic impact of cardiovascular disease hospitalization in four low- and middle-income countries. PLoS ONE 2011;6:e20821. 10.1371/journal.pone.0020821 21695127 PMC3114849

[9] Mszar R , Grandhi GR , Valero-Elizondo J , et al . Cumulative burden of financial hardship from medical bills across the spectrum of diabetes mellitus and atherosclerotic cardiovascular disease among non-elderly adults in the United States. J Am Heart Assoc 2020;9:e015523. 10.1161/JAHA.119.015523 32394783 PMC7660844

[10] Miller J , Edwards LD , Agustí A , et al . Comorbidity, systemic inflammation and outcomes in the ECLIPSE cohort. Respir Med 2013;107:1376–84. 10.1016/j.rmed.2013.05.001 23791463

[11] Haffner SM . Relationship of metabolic risk factors and development of cardiovascular disease and diabetes. Obesity (Silver Spring) 2006;14 Suppl 3:121S–127S. 10.1038/oby.2006.291 16931493

[12] Buddeke J , Bots ML , van Dis I , et al . Trends in comorbidity in patients hospitalised for cardiovascular disease. Int J Cardiol 2017;248:382–8. 10.1016/j.ijcard.2017.06.106 28712563

[13] De Hert M , Detraux J , Vancampfort D . The intriguing relationship between coronary heart disease and mental disorders. Dialogues Clin Neurosci 2018;20:31–40. 10.31887/DCNS.2018.20.1/mdehert 29946209 PMC6016051

[14] Hansen S . Mental health issues associated with cardiovascular disease in women. Psychiatr Clin North Am 2003;26:693–712. 10.1016/s0193-953x(03)00037-6 14563104

[15] Huang K-L , Su T-P , Chen T-J , et al . Comorbidity of cardiovascular diseases with mood and anxiety disorder: a population based 4-year study. Psychiatry Clin Neurosci 2009;63:401–9. 10.1111/j.1440-1819.2009.01974.x 19566773

[16] Möller-Leimkühler AM . Gender differences in cardiovascular disease and comorbid depression. Dialogues Clin Neurosci 2007;9:71–83. 10.31887/DCNS.2007.9.1/ammoeller 17506227 PMC3181845

[17] McGill HC , McMahan CA , Gidding SS . Preventing heart disease in the 21st century: implications of the pathobiological determinants of Atherosclerosis in youth (PDAY) study. Circulation 2008;117:1216–27. 10.1161/CIRCULATIONAHA.107.717033 18316498

[18] Karunathilake SP , Ganegoda GU . Secondary prevention of cardiovascular diseases and application of technology for early diagnosis. Biomed Res Int 2018;2018:5767864. 10.1155/2018/5767864 29854766 PMC5964583

[19] Muhammad Y , Almoteri M , Mujlid H , et al . An ML-enabled Internet of things framework for early detection of heart disease. Biomed Res Int 2022;2022:12. 10.1155/2022/3372296 PMC9519282 36187499

[20] Muhammad Y , Tahir M , Hayat M , et al . Early and accurate detection and diagnosis of heart disease using intelligent computational model. Sci Rep 2020;10:19747. 10.1038/s41598-020-76635-9 33184369 PMC7665174

[21] Mensah GA , Wei GS , Sorlie PD , et al . Decline in cardiovascular mortality: possible causes and implications. Circ Res 2017;120:366–80. 10.1161/CIRCRESAHA.116.309115 28104770 PMC5268076

[22] Ergin A , Muntner P , Sherwin R , et al . Secular trends in cardiovascular disease mortality, incidence, and case fatality rates in adults in the United States. Am J Med 2004;117:219–27. 10.1016/j.amjmed.2004.03.017 15308430

[23] Gilthorpe MS , Wilson RC . Rural/urban differences in the association between deprivation and healthcare utilisation. Soc Sci Med 2003;57:2055–63. 10.1016/s0277-9536(03)00071-6 14512237

[24] Farmer J , Iversen L , Campbell NC , et al . Rural/urban differences in accounts of patients' initial decisions to consult primary care. Health Place 2006;12:210–21. 10.1016/j.healthplace.2004.11.007 16338636

[25] Kaufman EA , McDonell MG , Cristofalo MA , et al . Exploring barriers to primary care for patients with severe mental illness: frontline patient and provider accounts. Issues Ment Health Nurs 2012;33:172–80. 10.3109/01612840.2011.638415 22364429

[26] Levinson Miller C , Druss BG , Dombrowski EA , et al . Barriers to primary medical care among patients at a community mental health center. Psychiatr Serv 2003;54:1158–60. 10.1176/appi.ps.54.8.1158 12883146

[27] Ross LE , Vigod S , Wishart J , et al . Barriers and facilitators to primary care for people with mental health and/or substance use issues: a qualitative study. BMC Fam Pract 2015;16:135. 10.1186/s12875-015-0353-3 26463083 PMC4604001

[28] Klersy C , De Silvestri A , Gabutti G , et al . A meta-analysis of remote monitoring of heart failure patients. J Am Coll Cardiol 2009;54:1683–94. 10.1016/j.jacc.2009.08.017 19850208

[29] Kulshreshtha A , Kvedar JC , Goyal A , et al . Use of remote monitoring to improve outcomes in patients with heart failure: a pilot trial. Int J Telemed Appl 2010;2010:870959. 10.1155/2010/870959 20508741 PMC2874922

[30] Best J . Wearable technology: COVID-19 and the rise of remote clinical monitoring. BMJ 2021;372:413. 10.1136/bmj.n413 33602670

[31] Chidambaram S , Erridge S , Kinross J , et al . Observational study of UK mobile health apps for COVID-19. Lancet Digit Health 2020;2:e388–90. 10.1016/S2589-7500(20)30144-8 32835196 PMC7314454

[32] Powell J , Newhouse N , Boylan A-M , et al . Digital health citizens and the future of the NHS. Digit Health 2016;2:2055207616672033. 10.1177/2055207616672033 29942569 PMC6001209

[33] Islam SMS , Maddison R . Digital health approaches for cardiovascular diseases prevention and management: lessons from preliminary studies. Mhealth 2021;7:41. 10.21037/mHealth-2020-6 34345618 PMC8326947

[34] Zwack CC , Haghani M , Hollings M , et al . The evolution of digital health technologies in cardiovascular disease research. NPJ Digit Med 2023;6:1. 10.1038/s41746-022-00734-2 36596833 PMC9808768

[35] Whitelaw S , Pellegrini DM , Mamas MA , et al . Barriers and facilitators of the uptake of digital health technology in cardiovascular care: a systematic scoping review. Eur Heart J Digit Health 2021;2:62–74. 10.1093/ehjdh/ztab005 34048508 PMC8139413

[36] Bhavnani SP . Digital health: opportunities and challenges to develop the next-generation technology-enabled models of cardiovascular care. Methodist Debakey Cardiovasc J 2020;16:296–303. 10.14797/mdcj-16-4-296 33500758 PMC7812856

[37] Dagher L , Nedunchezhian S , El Hajjar AH , et al . A cardiovascular clinic patients' survey to assess challenges and opportunities of digital health adoption during the COVID-19 pandemic. Cardiovasc Digit Health J 2022;3:31–9. 10.1016/j.cvdhj.2021.10.007 34812430 PMC8600804

[38] Gilbey D , Morgan H , Lin A , et al . Effectiveness, acceptability, and feasibility of Digital health interventions for LGBTIQ+ young people: systematic review. J Med Internet Res 2020;22:e20158. 10.2196/20158 33270039 PMC7746499

[39] Gentili C , Zetterqvist V , Rickardsson J , et al . Actsmart – development and feasibility of digital acceptance and commitment therapy for adults with chronic pain. NPJ Digit Med 2020;3:20. 10.1038/s41746-020-0228-4 32128450 PMC7018849

[40] Braun V , Clarke V . Using thematic analysis in psychology. Qual Res Psychol 2006;3:77–101. 10.1191/1478088706qp063oa

[41] Tong A , Sainsbury P , Craig J . Consolidated criteria for reporting qualitative research (COREQ): a 32-item checklist for interviews and focus groups. Int J Qual Health Care 2007;19:349–57. 10.1093/intqhc/mzm042 17872937

