# Supplementary material for: Stakeholder-led understanding of the implementation of digital technologies within heart disease diagnosis: a qualitative study protocol
Source: BMJ Open. 2023 Jun 27;13(6):e072952. doi: 10.1136/bmjopen-2023-072952 (PMC10410804; doi:10.1136/bmjopen-2023-072952)
Supplement: Author's manuscript [file bmjopen-2023-072952.draft_revisions.pdf]

BMJ Open

BMJ Open is committed to open peer review. As part of this commitment we make the peer review history of every article we publish publicly available.

When an article is published we post the peer reviewers' comments and the authors' responses online. We also post the versions of the paper that were used during peer review. These are the versions that the peer review comments apply to.

The versions of the paper that follow are the versions that were submitted during the peer review process. They are not the versions of record or the final published versions. They should not be cited or distributed as the published version of this manuscript.

BMJ Open is an open access journal and the full, final, typeset and author-corrected version of record of the manuscript is available on our site with no access controls, subscription charges or pay-per-view fees (http://bmjopen.bmj.com).

If you have any questions on BMJ Open's open peer review process please email info.bmjopen@bmj.com

BMJ Open

A stakeholder-led understanding of the implementation of digital technologies within heart disease diagnosis: a qualitative study protocol

Journal:	*BMJ Open*
Manuscript ID	bmjopen-2023-072952
Article Type:	Protocol
Date Submitted by the Author:	20-Feb-2023
Complete List of Authors:	Abdullayev, Kamilla; University of Sussex, School of Psychology<br>Chico, Timothy; The University of Sheffield, Department of Infection, Immunity and Cardiovascular Disease, The Medical School<br>Manktelow, Matthew; University of Ulster, School of Computing, Engineering and Intelligent Systems<br>Buckley, Oliver; University of East Anglia, 4. School of Computing Sciences<br>Condell, Joan; University of Ulster, School of Computing, Engineering and Intelligent Systems<br>Van Arkel, Richard; Imperial College London, Department of Mechanical Engineering<br>Diaz, Vanessa; University College London, Department of Mechanical Engineering; University College London, Wellcome/EPSRC Centre for Interventional and Surgical Sciences<br>Matcham, Faith; University of Sussex, School of Psychology
Keywords:	Adult cardiology < CARDIOLOGY, MENTAL HEALTH, PREVENTIVE MEDICINE

SCHOLARONE™
Manuscripts

A STAKEHOLDER-LED UNDERSTANDING OF THE IMPLEMENTATION OF DIGITAL TECHNOLOGIES WITHIN HEART DISEASE DIAGNOSIS: A QUALITATIVE STUDY PROTOCOL

Abdullayev K[1], Chico TJA[2], Manktelow M[3], Buckley O[4], Condell J[3], Van Arkel RJ[5], Diaz V[6,7], Dr Faith Matcham[1]

Corresponding Author: Kamilla Abdullayev, kga21@sussex.ac.uk

Affiliations:

1. *School of Psychology, University of Sussex, Brighton, UK*
2. *Department of Infection, Immunity and Cardiovascular Disease, The Medical School, University of Sheffield, Sheffield, UK*
3. *School of Computing, Engineering and Intelligent Systems, Ulster University at Magee, Londonderry, UK.*
4. *School of Computing Sciences, University of East Anglia, Norwich, UK*
5. *Department of Mechanical Engineering, Imperial College London, London, UK.*
6. *Department of Mechanical Engineering, University College London, London, UK.*
7. *Wellcome/EPSRC Centre for Interventional and Surgical Sciences, University College London, London, UK.*

Abstract

Introduction Cardiovascular diseases are highly prevalent amongst the UK population, and the quality of care is being reduced due to accessibility and resource issues. Increased implementation of digital technologies into the cardiovascular care pathway has enormous potential to lighten the load on the NHS, however, it is not possible to adopt this shift without embedding the perspectives of service users and clinicians.

Methods and Analysis A series of qualitative studies will be carried out with the aim of developing a stakeholder-led perspective on the implementation of digital technologies to improve holistic diagnosis of heart disease. Focus groups will be carried out with 20 people with lived experience of heart disease, and interviews will be carried out with 10 clinicians with experience of diagnosing heart diseases. The data will be analysed using an inductive thematic analysis approach.

For peer review only

Ethics and Dissemination This study received ethical approval from the Sciences and Technology Cross Research Council at the University of Sussex. The findings will be disseminated through conference presentations, peer-reviewed publications and to the study participants.

Key words: Adult Cardiology, Mental Health, Preventative Medicine

Article Summary:

Strengths and Limitations of this study

- The study materials have been informed by patient advisory boards, meaning they are sensitive to the experiences of the participants and the clinicians that will be recruited.
- The study will allow an in-depth understanding of the attitudes and experience of people with lived experience of heart disease and clinicians with experience of diagnosing heart disease.
- The use of an online research platform for participant recruitment will disadvantage those less comfortable using online resources. This may include those living in deprived areas without access to high quality technologies (which disproportionately excludes ethnic minority groups) and clinical populations with comorbidities that prevent the use of certain technologies, such as visual, auditory, physical, or learning impairments.
- The use of thematic analysis will not be free from the influence of the researcher's personal experience and knowledge

For peer review only

Introduction

Cardiovascular diseases (CVDs) are the leading cause of global deaths and are highly prevalent across the world (1). The situation in the UK is no different, with the British Heart Foundation reporting 7.6 million people living in the UK with a heart and circulatory disease in August 2022, and approximately 460 deaths a day (2). In addition to debilitating cardiovascular symptoms, individuals suffering from CVDs often experience a diminished quality of life (3–5), financial burdens from medication and treatments (6–9), and both physical (10–12) and psychological (13–16) comorbidities.

Although CVDs are highly prevalent and potentially very damaging, they can become considerably more manageable, or even preventable, if diagnosed early enough (17). Therefore, implementing secondary prevention measures, in the form of early diagnosis and interventions, is the most effective way of reducing the life-threatening or long-term impacts of CVDs on patient health and wellbeing (18). Technological advancements within artificial intelligence and data mining may provide the tools for more accurate and effective diagnosis (19,20), and can contribute to reducing cardiovascular mortality (21,22).

In addition to facilitating earlier and more accurate detection of CVDs through artificial intelligence, the increased use of "wearable" ambulatory assessment technology within healthcare has provided solutions for groups facing barriers that are preventing access to primary care, such as transportation limitations in remote locations (23,24), or reduced mobility or capacity due to mental illness (25–27). As a result, those who would have missed the opportunity to receive a timely diagnosis and appropriate treatment, are no longer being excluded due to logistical limitations. Overall, it is clear that the use of remote monitoring technology in heart disease can increase the likelihood of survival and decrease the burden of CVD on the individual and ultimately on healthcare services (28,29).

A variety of digital tools that allow more remote healthcare are already integrated into the NHS. For example, patient records can be accessed remotely thanks to the use electronic databases; patients' heart rhythms can be monitored over 24 hours from their homes using Holter monitors; and many appointments are carried out on videoconferencing software or

For peer review only

telephone calls. Although there is common complaint regarding the quality and modernity of technology being used within the NHS, it is evident – particularly since the COVID-19 pandemic (30,31) – that there has been a rapid increase in the adoption of digital technology within both the NHS (32) and across the world (33). Accordingly, there has also been increased research activity investigating the use of digital technologies in cardiovascular health (34).

When examining the growing use of digital health technologies, it is important to highlight the distinction between the types of technologies that are being implemented, specifically between medical grade devices and consumer technologies. Medical-grade devices require a specific regulatory approval to be used and can come in many forms, including implanted defibrillators and pacemakers, and 24-hour electrocardiograms. The data collected by these devices is usually physiological and requires specialists to monitor and interpret. On the other hand, consumer technologies can include health apps on our smartphones which track our sleep, step-count, and medication adherence. These types of technologies are usually in the control of the individual, allowing self-monitoring of symptoms and self-management of many conditions, meaning there is less dependence on an already-overwhelmed healthcare system. Therefore, when the increased accuracy of data produced by medical-grade devices is combined with the detailed and personalised information collected by lifestyle technologies, it allows for a more holistic account of patient health to facilitate an accurate and efficient diagnosis of potential heart diseases.

Nevertheless, along with the potential for improving the accuracy and efficiency of diagnosis, the implementation of digital technologies into healthcare poses challenges with usability, feasibility, and accessibility for patients (35–37), particularly those from under-represented groups in society (38,39). Too often, devices and systems are created without enough information regarding the patients' and clinicians' needs, which ultimately results in poor engagement and low cost-effectiveness (35). This highlights the importance of using the voices of stakeholders to inform the development and dissemination of digital health tools into cardiovascular care, as well as any other field within healthcare, otherwise the likelihood of positive effects is very low.

For peer review only

Therefore, a key factor in the growing shift towards the use of digital health tools within any care pathway is to gain a deeper understanding into how the new technology will fit into the existing systems, and to consider the barriers or facilitators to positive engagement from both patients and clinicians.

Study Objectives

The aim of this project is to develop a stakeholder-led understanding of the implementation of digital technologies to improve holistic diagnosis of heart disease. We have set the following three objectives which will be met via a series of qualitative studies with clinicians and individuals with lived experience of heart disease:

1) Develop a deeper understanding of stakeholder experiences of heart disease diagnosis
2) Understand stakeholder perspectives on the use of digital health tools within the heart disease diagnosis process
3) Explore the most meaningful and useful way of relaying digital data back to patients and clinicians

Methods & Analyses

Patient and Public Involvement

Prior to the starting participant recruitment, this research was reviewed by a cardiovascular patient advisory group based in Sheffield, which involved all participant-facing documents, including the recruitment materials and focus group schedules. This means we can be confident that the language we will use in the focus groups will be accessible and easy to understand, as well as ensuring that we are covering the important aspects of patient experience during diagnosis and when being asked to use digital health tools by their healthcare providers.

We also met with the NIHR Maudsley Biomedical Research Centre's Race, Ethnicity and Diversity (READ) advisory group to discuss the implementation of digital technologies into heart disease diagnosis from a cultural and ethnicity perspective. This meeting highlighted

For peer review only

the importance of considering how cultural and religious attitudes would affect engagement with digital technologies aiming to collect health data, as well as emphasising the role of family in individuals' healthcare amongst ethnic minorities. Given the potential exclusion of certain populations because of our recruitment method, this insight will provide a deeper understanding of additional factors that might contribute to the acceptance and engagement of digital technologies aimed to improve holistic diagnosis of heart disease.

Study Design

This will be a qualitative study, which will use both focus group and interview designs. The topic guides were developed based on the study objectives, with an even split between (1) patient/clinician experiences of diagnosis and (2) perspectives on the use of digital technologies within healthcare.

Study Population

For the focus groups, a target of 20 individuals with lived experience of heart disease will be enrolled in total. The following inclusion/exclusion criteria will be applied:
Inclusion criteria: Lifetime diagnosis of heart disease (including but not limited to: angina, heart failure, valve disease, abnormal heart rhythms); aged 18 or over; able to speak English at a level sufficient for participation; able to give informed consent for participation.
Exclusion criteria: Major cognitive impairment or dementia preventing participation.

For the interviews, a target of 10 clinicians who have had any experience (past or present) of diagnosing people with heart disease will be enrolled. The eligibility criteria for inclusion are at least 6 months of experience in working with heart disease patients in any capacity (including cardiology, nursing, primary care, or other healthcare professional in multidisciplinary teams); aged 18 or over; able to speak fluent English; and able to give informed consent for participation.

Procedure

As we are interested in hearing from participants across a range of disease durations (ie, those with very early symptoms and those who are on established management plans. We plan to recruit via: social media platforms such as LinkedIn, Twitter and Instagram; Prolific;

For peer review only

and existing cohorts of people from the investigators' previous research studies who have consented to be contacted for research purposes. Clinicians will be recruited using purposive sampling via personal and professional connections, and social media platforms such as LinkedIn, Twitter and Instagram.

All focus groups and interviews will be carried out online over Zoom, and consent and baseline demographic data will also be collected via online Qualtrics surveys prior to the online study. We expect focus groups to take about two hours and interviews to take about one hour. The focus groups and interviews will be semi-structured and will follow a pre-approved question schedule, split into two sections – patient experience of heart disease and views on digital technologies within healthcare (see Appendix). The same researcher will facilitate all the sessions.

Data Analysis Plan

Descriptive statistics for demographics, current mental distress levels, confidence using technologies, and participant type-specific questions (including length of time in clinical role for clinicians and details on health condition for participants with lived-experience of heart disease) will be presented.

Transcriptions from both focus group and interview recordings will be validated by the team of researchers and coded and analysed using the NVivo software. In line with Braun and Clarke's 2006 recommendations (40), an inductive thematic analysis approach will be taken, whereby the data from the transcripts will decide the themes, instead of basing them on any previous theoretical basis.

Ethics and Dissemination

This study was reviewed and approved by the Sciences & Technology Cross-School Research Ethics Council at the University of Sussex (reference ER/FM409/1). The findings will be disseminated through conference presentations, peer-reviewed publications and to the study participants.

For peer review only

Acknowledgements:

We would like to thank the two patient and public involvement groups that helped to inform the design of this study: The NIHR Maudsley Biomedical Research Centre's Race, Ethnicity and Diversity (READ) advisory group and the Sheffield-based Cardiology Patient group.

Author Statement:

Conceptualisation: F Matcham, TJ Chico, J Condell, O Buckley, V Diaz, R Van Arkel
Methodology: F Matcham, TJ Chico
Investigation: K Abdullayev
Writing – original draft: K Abdullayev
Writing – review and editing: F Matcham, T.J Chico
Supervision: F Matcham, TJ Chico
Project Administration: K Abdullayev, F Matcham
Funding Acqusition: F Matcham, TJ Chico, J Condell, O Buckley, V Diaz, R Van Arkel

Funding Statement:

This work is supported by the UK Engineering and Physical Sciences Research Council (ESPRC) (grant number: EP/X000257/1).

Competing Interests Statement:

There are no conflicts of interest

Word Count:

1564

For peer review only

References

1. World Health Organisation (WHO). Cardiovascular diseases (CVDs) [Internet]. 2011 [cited 2023 Feb 3]. Available from: https://www.who.int/news-room/fact-sheets/detail/cardiovascular-diseases-(cvds)
2. Health Intelligence Team B. Our vision is a world free from the fear of heart and circulatory diseases. UK Factsheet. 2022.
3. Stafford M, Soljak M, Pledge V, Mindell J. Socio-economic differences in the health-related quality of life impact of cardiovascular conditions. Eur J Public Health [Internet]. 2012 Jun 1 [cited 2023 Feb 3];22(3):301–5. Available from: https://academic.oup.com/eurpub/article/22/3/301/505231
4. Pandya A, Gaziano TA, Weinstein MC, Cutler D. More americans living longer with cardiovascular disease will increase costs while lowering quality of life. Health Aff. 2013 Aug 2;32(10):1706–14.
5. Tušek-Bunc K, Petek D. Comorbidities and characteristics of coronary heart disease patients: Their impact on health-related quality of life. Health Qual Life Outcomes. 2016;14(1).
6. Annapureddy A, Valero-Elizondo J, Khera R, Grandhi GR, Spatz ES, Dreyer RP, et al. Association Between Financial Burden, Quality of Life, and Mental Health Among Those With Atherosclerotic Cardiovascular Disease in the United States. Circ Cardiovasc Qual Outcomes [Internet]. 2018 Nov 1 [cited 2023 Feb 3];11(11):e005180. Available from: https://www.ahajournals.org/doi/abs/10.1161/CIRCOUTCOMES.118.005180
7. Li C, Young BR, Jian W. Association of socioeconomic status with financial burden of disease among elderly patients with cardiovascular disease: Evidence from the China Health and Retirement Longitudinal Survey. BMJ Open [Internet]. 2018 [cited 2023 Feb 3];8(3):18703. Available from: http://bmjopen.bmj.com/
8. Huffman MD, Rao KD, Pichon-Riviere A, Zhao D, Harikrishnan S, Ramaiya K, et al. A cross-sectional study of the microeconomic impact of cardiovascular disease hospitalization in four low- and middle-income countries. PLoS One [Internet]. 2011 [cited 2023 Feb 3];6(6):e20821. Available from: https://journals.plos.org/plosone/article?id=10.1371/journal.pone.0020821

For peer review only

9. Mszar R, Grandhi GR, Valero-Elizondo J, Caraballo C, Khera R, Desai N, et al. Cumulative burden of financial hardship from medical bills across the spectrum of diabetes mellitus and atherosclerotic cardiovascular disease among non-elderly adults in the United States. J Am Heart Assoc [Internet]. 2020 May 18 [cited 2023 Feb 3];9(10):15523. Available from: https://www.ahajournals.org/doi/abs/10.1161/JAHA.119.015523

10. Miller J, Edwards LD, Agustí A, Bakke P, Calverley PMA, Celli B, et al. Comorbidity, systemic inflammation and outcomes in the ECLIPSE cohort. Respir Med. 2013 Sep 1;107(9):1376–84.

11. Haffner SM. Relationship of metabolic risk factors and development of cardiovascular disease and diabetes. [Internet]. Vol. 14 Suppl 3, Obesity (Silver Spring, Md.). John Wiley & Sons, Ltd; 2006 [cited 2023 Feb 3]. p. 121S-127S. Available from: https://onlinelibrary.wiley.com/doi/full/10.1038/oby.2006.291

12. Buddeke J, Bots ML, van Dis I, Liem A, Visseren FLJ, Vaartjes I. Trends in comorbidity in patients hospitalised for cardiovascular disease. Int J Cardiol. 2017 Dec 1;248:382–8.

13. De Hert M, Detraux J, Vancampfort D. The intriguing relationship between coronary heart disease and mental disorders. Dialogues Clin Neurosci [Internet]. 2018 Mar 1 [cited 2023 Feb 3];20(1):31–40. Available from: https://www.tandfonline.com/doi/abs/10.31887/DCNS.2018.20.1/mdehert

14. Hansen S. Mental health issues associated with cardiovascular disease in women [Internet]. Vol. 26, Psychiatric Clinics of North America. Elsevier; 2003 [cited 2023 Feb 3]. p. 693–712. Available from: http://www.psych.theclinics.com/article/S0193953X03000376/fulltext

15. Huang KL, Su TP, Chen TJ, Chou YH, Bai YM. Comorbidity of cardiovascular diseases with mood and anxiety disorder: A population based 4-year study. Psychiatry Clin Neurosci [Internet]. 2009 Jun 1 [cited 2023 Feb 3];63(3):401–9. Available from: https://onlinelibrary.wiley.com/doi/full/10.1111/j.1440-1819.2009.01974.x

16. Möller-Leimkühler AM. Gender differences in cardiovascular disease and comorbid depression. Dialogues Clin Neurosci [Internet]. 2007 [cited 2023 Feb 3];9(1):71–83. Available from: https://www.tandfonline.com/doi/abs/10.31887/DCNS.2007.9.1/ammoeller

For peer review only

17. McGill HC, McMahan CA, Gidding SS. Preventing heart disease in the 21st century: Implications of the pathobiological determinants of atherosclerosis in youth (PDAY) study [Internet]. Vol. 117, Circulation. Lippincott Williams & Wilkins; 2008 [cited 2023 Feb 3]. p. 1216–27. Available from: https://www.ahajournals.org/doi/abs/10.1161/CIRCULATIONAHA.107.717033

18. Karunathilake SP, Ganegoda GU. Secondary Prevention of Cardiovascular Diseases and Application of Technology for Early Diagnosis. Martinez E, editor. Biomed Res Int [Internet]. 2018;2018:5767864. Available from: https://doi.org/10.1155/2018/5767864

19. Muhammad Y, Almoteri M, Mujlid H, Alharbi A, Alqurashi F, Dutta AK, et al. An ML-Enabled Internet of Things Framework for Early Detection of Heart Disease. Biomed Res Int [Internet]. 2022 [cited 2023 Feb 3];2022:12. Available from: https://doi.org/10.1155/2022/3372296

20. Muhammad Y, Tahir M, Hayat M, Chong KT. Early and accurate detection and diagnosis of heart disease using intelligent computational model. Sci Rep [Internet]. 2020 [cited 2023 Feb 3];10(1). Available from: https://doi.org/10.1038/s41598-020-76635-9

21. Mensah GA, Wei GS, Sorlie PD, Fine LJ, Rosenberg Y, Kaufmann PG, et al. Decline in Cardiovascular Mortality: Possible Causes and Implications [Internet]. Vol. 120, Circulation Research. Lippincott Williams & Wilkins Hagerstown, MD; 2017 [cited 2023 Feb 3]. p. 366–80. Available from: https://www.ahajournals.org/doi/abs/10.1161/CIRCRESAHA.116.309115

22. Ergin A, Muntner P, Sherwin R, He J. Secular trends in cardiovascular disease mortality, incidence, and case fatality rates in adults in the United States. Am J Med. 2004 Aug 15;117(4):219–27.

23. Gilthorpe MS, Wilson RC. Rural/urban differences in the association between deprivation and healthcare utilisation. Soc Sci Med. 2003 Dec 1;57(11):2055–63.

24. Farmer J, Iversen L, Campbell NC, Guest C, Chesson R, Deans G, et al. Rural/urban differences in accounts of patients' initial decisions to consult primary care. Heal Place. 2006 Jun 1;12(2):210–21.

25. Kaufman EA, McDonell MG, Cristofalo MA, Ries RK. Exploring barriers to primary care for patients with severe mental illness: Frontline patient and provider accounts.

For peer review only

Issues Ment Health Nurs [Internet]. 2012 Mar [cited 2023 Feb 3];33(3):172–80. Available from: https://www.tandfonline.com/doi/abs/10.3109/01612840.2011.638415

26. Miller CL, Druss BG, Dombrowski EA, Rosenheck RA. Barriers to primary medical care among patients at a community mental health center. Psychiatr Serv [Internet]. 2003 Aug 1 [cited 2023 Feb 3];54(8):1158–60. Available from: https://ps.psychiatryonline.org/doi/10.1176/appi.ps.54.8.1158

27. Ross LE, Vigod S, Wishart J, Waese M, Spence JD, Oliver J, et al. Barriers and facilitators to primary care for people with mental health and/or substance use issues: A qualitative study. BMC Fam Pract. 2015;16(1).

28. Klersy C, De Silvestri A, Gabutti G, Regoli F, Auricchio A. A Meta-Analysis of Remote Monitoring of Heart Failure Patients. J Am Coll Cardiol. 2009 Oct 27;54(18):1683–94.

29. Kulshreshtha A, Kvedar JC, Goyal A, Halpern EF, Watson AJ. Use of remote monitoring to improve outcomes in patients with heart failure: A pilot trial. Int J Telemed Appl [Internet]. 2010 Jan 1 [cited 2023 Feb 3];2010. Available from: https://dl.acm.org/doi/10.1155/2010/870959

30. Best J. Wearable technology: Covid-19 and the rise of remote clinical monitoring [Internet]. Vol. 372, The BMJ. British Medical Journal Publishing Group; 2021 [cited 2023 Feb 3]. Available from: https://www.bmj.com/content/372/bmj.n413

31. Chidambaram S, Erridge S, Kinross J, Purkayastha S. Observational study of UK mobile health apps for COVID-19 [Internet]. Vol. 2, The Lancet Digital Health. Elsevier Ltd; 2020 [cited 2023 Feb 3]. p. e388–90. Available from: http://www.thelancet.com/article/S2589750020301448/fulltext

32. Powell J, Newhouse N, Boylan A-M, Williams V. Digital health citizens and the future of the NHS. Digit Heal [Internet]. 2016 [cited 2023 Feb 3];2:205520761667203. Available from: https://us.sagepub.com/en-us/nam/open-access-at-sage

33. Islam SMS, Maddison R. Digital health approaches for cardiovascular diseases prevention and management: Lessons from preliminary studies [Internet]. Vol. 7, mHealth. AME Publications; 2021 [cited 2023 Feb 3]. Available from: /pmc/articles/PMC8326947/

34. Zwack CC, Haghani M, Hollings M, Zhang L, Gauci S, Gallagher R, et al. The evolution of digital health technologies in cardiovascular disease research [Internet]. Vol. 6, npj

For peer review only

Digital Medicine. Nature Publishing Group; 2023 [cited 2023 Feb 3]. p. 1–11. Available from: https://www.nature.com/articles/s41746-022-00734-2

35. Whitelaw S, Pellegrini DM, Mamas MA, Cowie M, Van Spall HGC. Barriers and facilitators of the uptake of digital health technology in cardiovascular care: a systematic scoping review. Eur Hear J - Digit Heal [Internet]. 2021 May 4 [cited 2023 Feb 3];2(1):62–74. Available from: https://academic.oup.com/ehjdh/article/2/1/62/6128570

36. Bhavnani SP. Digital Health: Opportunities and Challenges to Develop the Next-Generation Technology-Enabled Models of Cardiovascular Care [Internet]. Vol. 16, Methodist DeBakey cardiovascular journal. Houston Methodist DeBakey Heart & Vascular Center; 2020 [cited 2023 Feb 3]. p. 296–303. Available from: https://journal.houstonmethodist.org/articles/10.14797/mdcj-16-4-296

37. Dagher L, Nedunchezhian S, El Hajjar AH, Zhang Y, Deffer O, Russell A, et al. A cardiovascular clinic patients' survey to assess challenges and opportunities of digital health adoption during the COVID-19 pandemic. Cardiovasc Digit Heal J [Internet]. 2022 Feb 1 [cited 2023 Feb 3];3(1):31–9. Available from: http://www.cvdigitalhealthjournal.com/article/S2666693621001249/fulltext

38. Gilbey D, Morgan H, Lin A, Perry Y. Effectiveness, acceptability, and feasibility of digital health interventions for LGBTIQ+ young people: Systematic review [Internet]. Vol. 22, Journal of Medical Internet Research. Journal of Medical Internet Research; 2020 [cited 2023 Feb 3]. p. e20158. Available from: https://www.jmir.org/2020/12/e20158

39. Gentili C, Zetterqvist V, Rickardsson J, Holmström L, Simons LE, Wicksell RK. ACTsmart – development and feasibility of digital Acceptance and Commitment Therapy for adults with chronic pain. npj Digit Med [Internet]. 2020 Feb 13 [cited 2023 Feb 3];3(1):1–12. Available from: https://www.nature.com/articles/s41746-020-0228-4

40. Braun V, Clarke V. Using thematic analysis in psychology. Qualitative research in psychology. 2006 Jan 1;3(2):77-101.

For peer review only

Digital Twin Question Schedules

Lived Experience Focus Group Questions

Heart Disease-related Questions (diagnosis process)

1. Please describe the series of events which took place that led to your heart disease diagnosis
   - Possible prompts: how involved did you feel in this process, active/passive, taken seriously by clinicians/dismissed, big/small role

2. How did you keep track of your symptoms before you were given your diagnosis?
   - Possible prompts: digital tools used, manual log, own method

3. After you first brought up concerns about your heart to your doctor, what did they ask you to do, if anything?
   - Possible prompts: digital tools used, manual log, own method

4. What types of numeric data did your doctor discuss with you, if any?
   - Possible prompts: percentage risks, heart rates, blood pressure, etc
   - Follow up: Was the meaning/importance of the data clear to you?

5. What other aspects of your overall health and wellbeing were important to you around the time you were getting diagnosed?
   - Possible prompts: existing comorbidities, mental health, social support, stress, financial status, lifestyle choices, health behaviours etc

6. During the diagnosis process, what were some important things/people that made the experience less difficult?
   - Possible prompts: staff, communication, social support at home

7. What were the greatest challenges for you during the diagnosis process?
   - Possible prompts: communication, access to information/emotional support, dealing with clinicians, difficulty understanding medical terminology

8. What were your experiences with doctors, nurses and other healthcare staff during the process of getting diagnosed?
   - Possible prompts: positive/negative experience, specific experiences standing out, communication, prejudices or discrimination?
   - Follow up (depending on previous responses) – how did this impact the rest of your treatment?
   - Follow up (depending on previous responses) – how has communication with your clinician impacted your treatment?

9. What do you feel delays or prevents people from getting accurate diagnoses?
   - Possible prompts: access to healthcare, language barriers, fear of stigma/discrimination, lack of concern for health, financial burdens, lack of social support

For peer review only

Digital Twin Question Schedules

10. How do you think we could address these things to help someone get a quicker and more accurate diagnosis?
    - Possible prompts: better, more objective data, easier to explain problems to clinicians, access to healthcare, language assistant, addressing stigmas, financial support, social/mental health/emotional support

Digital Technologies Questions

1. Technologies such as televisions, computers, smartphones etc. have become increasingly integral to our daily lives.
   How do you feel about using different types of technologies in your life?
   - Possible prompts: positive/negative, certain elements easier/harder

2. More specifically, digital technologies are those that can electronically generate, process and store different types of data. Digital technologies are increasingly being used in the healthcare sector.
   What experiences do you have using digital health tools within your healthcare treatment (doesn't have to be specific to heart disease diagnosis)?
   - Possible prompts: positive/negative, limited use, fit for purpose, implementation by clinicians, clinician experience with them

3. Thinking back to the challenges we mentioned in the first part of our discussions [give examples], do you feel digital technologies would help improve, or make worse, any of these issues? If so, which ones and how?
   - Note down issues mentioned and prompt those if needed

Digital health tools are being used more and more in the healthcare sector, as they can improve the way we diagnose and monitor many conditions (including heart disease) by tracking symptoms more effectively.

4. If you were given a digital health tool to help improve the accuracy of your heart disease diagnosis, what sort of things would put you off from using it?
   - Possible prompts: digital literacy, not seen as valuable, preference to deal with a real person, health status, financial status

5. What would motivate you to use it?
   - Possible prompts: incentives, feedback, avatar

6. How would you feel about getting feedback from this type of technology?
   - Follow up: how would it impact your engagement with it?
   - Possible prompts: agency in diagnosis, better awareness of condition/symptoms, more accurate monitoring

7. How would you feel about getting feedback from this device before seeing your doctor?
   - Possible prompts: examples like not enough movement, try to stand more etc;

For peer review only

Digital Twin Question Schedules

8. Who do you think should receive the information that comes from this device?
   - Prompts: clinician, patient, family

9. Do you think there will be any challenges when trying to include a digital monitoring tool in daily patient use?
   - Follow up: What factors do you think we need to consider to make it as easy to use as possible?

The final two questions will be more specific to the final product we are aiming to create. Try to imagine you have a wearable device that is linked to a smartphone app, which allows you to enter data about your symptoms, how you are feeling, certain stressors that may be occurring in your day and so on. This data would all be processed by the app and then reported back to you and your clinician in an easy-to-read format. Let's discuss what you think about specific elements that might be included…

10. If the data were to be sent to you, what would be the most helpful way for the data to be presented/communicated to you?
    - Possible prompts: features like colours, layout etc., how to communicate it – email summary, app interface etc, would you want to see it yourself before seeing your clinician or not?

11. What are your thoughts on having a virtual doctor/avatar/chatbot to interact with on a device?
    - Follow up: do you think being able to personalise the avatar would help with engagement and relatability of the virtual element?
    - Follow up: do you think this would be better than having a simple logging/tracking system like they use for other health tracking apps? Or more complicated?
    - Follow up: any immediate deterrents come to mind from having a 'chatbot' type function?

For peer review only

Digital Twin Question Schedules

Clinician Interview Questions

Heart Disease-related Questions (diagnosis process)

1. Please describe the series of events that normally take place when you diagnose a patient with heart disease.
   - Possible prompts: factors into your decision, length of time, with collaboration/advice from other colleagues, how it's changed with years of experience.

2. How do you monitor your patients' symptoms prior to/following diagnosis?
   - Follow up: what do you ask them to do?
   - Possible prompts: digital tools used, manual log, own method

3. Which numeric data do you find most valuable when making diagnoses?
   - Possible prompts: ECGs, heart rate, blood pressure etc.

4. What other aspects of overall patient health and wellbeing are important to you when you are making a heart disease diagnosis?
   - Possible prompts: existing comorbidities, mental health, social support, stress, financial status, lifestyle choices, health behaviours etc

5. What do you feel are important factors for ensuring a patient has a positive experience following a heart disease diagnosis?
   - Possible prompts: staff, communication, social support at home

6. What are the greatest challenges for you, as a clinician, during the diagnosis process?
   - Possible prompts: communication, medical advice adherence, language barriers, implicit biases due to potentially 'self-inflicted' nature of disease

7. What are your interactions with your patients like prior to/following their heart disease diagnosis?
   - Possible prompts: positive/negative experience, specific experiences standing out, communication, prejudices or discrimination?
   - Follow up (depending on previous responses) – how did this impact the rest of their treatment?
   - Follow up (depending on previous responses) – how has communication with your patients impacted your treatment?

8. What do you feel are potential barriers that prevent people from getting accurate diagnoses?
   - Possible prompts: access to healthcare, language barriers, fear of stigma/discrimination, lack of concern for health, financial burdens, lack of social support

For peer review only

Digital Twin Question Schedules

9. How do you think we could address these barriers to help someone get an accurate diagnosis faster?
   - Possible prompts: better, more objective data, easier to explain problems to clinicians, access to healthcare, language assistant, addressing stigmas, financial support, social/mental health/emotional support

Digital Technologies Questions

1. Technologies such as televisions, computers, smartphones etc. have become increasingly integral to our daily lives.
   How do you feel about using different types of technologies in your life?
   - Possible prompts: positive/negative, certain elements easier/harder

2. What experiences do you have using digital health tools within your healthcare profession? (doesn't have to be specific to heart disease diagnosis)
   - Possible prompts: positive/negative, limited use, fit for purpose, implementation by clinicians, clinician experience with them

3. Thinking back to the challenges we mentioned in the first part of our discussions [give examples], do you feel digital technologies would help improve, or make worse, any of these issues? If so, which ones and how?
   - Note down issues mentioned and prompt those if needed

As you may know, digital health tools are being used more and more in the healthcare sector, to improve the way we diagnose and monitor many conditions (including heart disease) by tracking symptoms more effectively.

4. What would prevent you from using/prescribing a digital health tool made for improving diagnosis of heart disease?
   - Possible prompts: digital literacy, not seen as valuable, preference to deal with a real person, health status, financial status

5. What do you think would prevent your patients from engaging with a digital health tool for symptom monitoring?
   - Possible prompts: digital literacy, not seen as valuable, preference to deal with a real person, health status, financial status

6. What would motivate you to engage in such technologies?
   - Possible prompts: incentives, feedback, avatar, certain data types/presentation/summaries

7. What do you think would motivate your patients to engage in such technologies?
   - Possible prompts: incentives, feedback, avatar

8. Do you feel that patients getting feedback from a digital monitoring system would improve engagement with it? If so, how, why/why not?
   - Possible prompts: agency in diagnosis, better awareness of condition/symptoms, more accurate monitoring

For peer review only

Digital Twin Question Schedules

9. How would you feel if your patients were getting feedback from the digital monitoring system before seeing you?
    - Possible prompts: examples like not enough movement, try to stand more etc;

10. Do you foresee any challenges with implementing a digital monitoring tool into daily patient use? What factors do you think we need to consider to make it as easy to use as possible?

The final two questions will be more specific to the final product we are aiming to create. Try to imagine you will be giving patients a wearable device that is linked to a smartphone app, which allows them to enter data about their symptoms, how they are feeling, certain stressors that may be occurring in their day and so on. This data would all be processed by the app and then reported back to you and your patient in an easy-to-read format. Let's discuss what you think about specific elements that might be included…

11. What are your thoughts on having a virtual doctor/avatar/chatbot to interact with on a device?
    - Follow up: do you think being able to personalise the avatar would help with engagement and relatability of the virtual element?
    - Follow up: do you think this would be better than having a simple logging/tracking system like they use for other health tracking apps? Or more complicated?
    - Follow up: any immediate deterrents come to mind from having a 'chatbot' type function?

12. What would be the most helpful way for the data to be presented/communicated to you?
    - Follow up: how do you think this should differ from how data is presented to patients, if at all?
    - Possible prompts: features like colours, layout etc., how to communicate it – email summary, app interface etc, would you want to see it yourself before seeing your clinician or not?

For peer review only

BMJ Open

A stakeholder-led understanding of the implementation of digital technologies within heart disease diagnosis: a qualitative study protocol

Journal:	*BMJ Open*
Manuscript ID	bmjopen-2023-072952.R1
Article Type:	Protocol
Date Submitted by the Author:	08-May-2023
Complete List of Authors:	Abdullayev, Kamilla; University of Sussex, School of Psychology<br>Chico, Timothy; The University of Sheffield, Department of Infection, Immunity and Cardiovascular Disease, The Medical School<br>Manktelow, Matthew; University of Ulster, School of Computing, Engineering and Intelligent Systems<br>Buckley, Oliver; University of East Anglia, 4. School of Computing Sciences<br>Condell, Joan; University of Ulster, School of Computing, Engineering and Intelligent Systems<br>Van Arkel, Richard; Imperial College London, Department of Mechanical Engineering<br>Diaz, Vanessa; University College London, Department of Mechanical Engineering; University College London, Wellcome/EPSRC Centre for Interventional and Surgical Sciences<br>Matcham, Faith; University of Sussex, School of Psychology
**Primary Subject Heading**:	Cardiovascular medicine
Secondary Subject Heading:	Patient-centred medicine
Keywords:	Adult cardiology < CARDIOLOGY, MENTAL HEALTH, PREVENTIVE MEDICINE

SCHOLARONE™
Manuscripts

A STAKEHOLDER-LED UNDERSTANDING OF THE IMPLEMENTATION OF DIGITAL TECHNOLOGIES WITHIN HEART DISEASE DIAGNOSIS: A QUALITATIVE STUDY PROTOCOL

Abdullayev K[1], Chico TJA[2], Manktelow M[3], Buckley O[4], Condell J[3], Van Arkel RJ[5], Diaz V[6,7], Dr Faith Matcham[1]

Corresponding Author: Kamilla Abdullayev, kga21@sussex.ac.uk

Affiliations:

1. *School of Psychology, University of Sussex, Brighton, UK*
2. *Department of Infection, Immunity and Cardiovascular Disease, The Medical School, University of Sheffield, Sheffield, UK*
3. *School of Computing, Engineering and Intelligent Systems, Ulster University at Magee, Londonderry, UK.*
4. *School of Computing Sciences, University of East Anglia, Norwich, UK*
5. *Department of Mechanical Engineering, Imperial College London, London, UK.*
6. *Department of Mechanical Engineering, University College London, London, UK.*
7. *Wellcome/EPSRC Centre for Interventional and Surgical Sciences, University College London, London, UK.*

For peer review only

Abstract

Introduction Cardiovascular diseases are highly prevalent amongst the UK population, and the quality of care is being reduced due to accessibility and resource issues. Increased implementation of digital technologies into the cardiovascular care pathway has enormous potential to lighten the load on the NHS, however, it is not possible to adopt this shift without embedding the perspectives of service users and clinicians.

Methods and Analysis

A series of qualitative studies will be carried out with the aim of developing a stakeholder-led perspective on the implementation of digital technologies to improve holistic diagnosis of heart disease. This will be a decentralised study with all data collection being carried out online with a nationwide cohort. Four focus groups, each with 5-6 participants, will be carried out with people with lived experience of heart disease, and 10 one-to-one

interviews will be carried out with clinicians with experience of diagnosing heart diseases. The data will be analysed using an inductive thematic analysis approach.

Ethics and Dissemination This study received ethical approval from the Sciences and Technology Cross Research Council at the University of Sussex (reference ER/FM409/1). Participants will be required to provide informed consent via a Qualtrics survey before being accepted into the online interview or focus group. The findings will be disseminated through conference presentations, peer-reviewed publications and to the study participants.

Key words: Adult Cardiology, Mental Health, Preventative Medicine

Article Summary:

Strengths and Limitations of this study

- The study materials have been informed by patient advisory boards, meaning they are sensitive to the experiences of the participants and the clinicians that will be recruited.
- The study will allow an in-depth understanding of the attitudes and experience of people with lived experience of heart disease and clinicians with experience of diagnosing heart disease.
- The use of an online research platform for participant recruitment will disadvantage certain demographic and clinical groups who are less comfortable using online resources.
- The use of thematic analysis will not be free from the influence of the researcher's personal experience and knowledge

For peer review only

Introduction

Cardiovascular diseases (CVDs) are the leading cause of global deaths and are highly prevalent across the world (1). The situation in the UK is no different, with the British Heart Foundation reporting 7.6 million people living in the UK with a heart and circulatory disease in August 2022, and approximately 460 deaths a day (2). In addition to debilitating cardiovascular symptoms, individuals suffering from CVDs often experience a diminished quality of life (3–5), financial burdens from medication and treatments (6–9), and both physical (10–12) and psychological (13–16) comorbidities.

Although CVDs are highly prevalent and potentially very damaging, they can become considerably more manageable, or even preventable, if diagnosed early enough (17). Therefore, implementing secondary prevention measures, in the form of early diagnosis and interventions, is the most effective way of reducing the life-threatening or long-term impacts of CVDs on patient health and wellbeing (18). Technological advancements within artificial intelligence and data mining may provide the tools for more accurate and effective diagnosis (19,20), and can contribute to reducing cardiovascular mortality (21,22).

In addition to facilitating earlier and more accurate detection of CVDs through artificial intelligence, the increased use of “wearable” ambulatory assessment technology within healthcare has provided solutions for groups facing barriers that are preventing access to primary care, such as transportation limitations in remote locations (23,24), or reduced mobility or capacity due to mental illness (25–27). As a result, those who would have missed the opportunity to receive a timely diagnosis and appropriate treatment, are no longer being excluded due to logistical limitations. Overall, it is clear that the use of remote monitoring technology in heart disease can increase the likelihood of survival and decrease the burden of CVD on the individual and ultimately on healthcare services (28,29).

A variety of digital tools that allow more remote healthcare are already integrated into the NHS. For example, patient records can be accessed remotely thanks to the use electronic databases; patients’ heart rhythms can be monitored over 24 hours from their homes using Holter monitors; and many appointments are carried out on videoconferencing software or

For peer review only

telephone calls. Although there is common complaint regarding the quality and modernity of technology being used within the NHS, it is evident – particularly since the COVID-19 pandemic (30,31) – that there has been a rapid increase in the adoption of digital technology within both the NHS (32) and across the world (33). Accordingly, there has also been increased research activity investigating the use of digital technologies in cardiovascular health (34).

When examining the growing use of digital health technologies, it is important to highlight the distinction between the types of technologies that are being implemented, specifically between medical grade devices and consumer technologies. Medical-grade devices require a specific regulatory approval to be used and can come in many forms, including implanted defibrillators and pacemakers, and 24-hour electrocardiograms. The data collected by these devices is usually physiological and requires specialists to monitor and interpret. On the other hand, consumer technologies can include health apps on our smartphones which track our sleep, step-count, and medication adherence. These types of technologies are usually in the control of the individual, allowing self-monitoring of symptoms and self-management of many conditions, meaning there is less dependence on an already-overwhelmed healthcare system. Therefore, when the increased accuracy of data produced by medical-grade devices is combined with the detailed and personalised information collected by lifestyle technologies, it allows for a more holistic account of patient health to facilitate an accurate and efficient diagnosis of potential heart diseases.

For peer review only

Nevertheless, along with the potential for improving the accuracy and efficiency of diagnosis, the implementation of digital technologies into healthcare poses challenges with usability, feasibility, and accessibility for patients (35–37), particularly those from under-represented groups in society (38,39). Too often, devices and systems are created without enough information regarding the patients' and clinicians' needs, which ultimately results in poor engagement and low cost-effectiveness (35). This highlights the importance of using the voices of stakeholders to inform the development and dissemination of digital health tools into cardiovascular care, as well as any other field within healthcare, otherwise the likelihood of positive effects is very low.

For peer review only - http://bmjopen.bmj.com/site/about/guidelines.xhtml

Therefore, a key factor in the growing shift towards the use of digital health tools within any care pathway is to gain a deeper understanding into how the new technology will fit into the existing systems, and to consider the barriers or facilitators to positive engagement from both patients and clinicians.

Study Objectives

The aim of this project is to develop a stakeholder-led understanding of the implementation of digital technologies to improve holistic diagnosis of heart disease. We have set the following three objectives which will be met via a series of qualitative studies with clinicians and individuals with lived experience of heart disease:

1) Develop a deeper understanding of stakeholder experiences of heart disease diagnosis
2) Understand stakeholder perspectives on the use of digital health tools within the heart disease diagnosis process
3) Explore the most meaningful and useful way of relaying digital data back to patients and clinicians

Methods & Analyses

Patient and Public Involvement

Prior to the starting participant recruitment, this research was reviewed by a cardiovascular patient advisory group based in Sheffield, which involved all participant-facing documents, including the recruitment materials and focus group schedules. This means we can be confident that the language we will use in the focus groups will be accessible and easy to understand, as well as ensuring that we are covering the important aspects of patient experience during diagnosis and when being asked to use digital health tools by their healthcare providers.

We also met with the NIHR Maudsley Biomedical Research Centre's Race, Ethnicity and Diversity (READ) advisory group to discuss the implementation of digital technologies into heart disease diagnosis from a cultural and ethnicity perspective. This meeting highlighted

For peer review only - http://bmjopen.bmj.com/site/about/guidelines.xhtml

the importance of considering how cultural and religious attitudes would affect engagement with digital technologies aiming to collect health data, as well as emphasising the role of family in individuals' healthcare amongst ethnic minorities. Given the potential exclusion of certain populations because of our recruitment method, this insight will provide a deeper understanding of additional factors that might contribute to the acceptance and engagement of digital technologies aimed to improve holistic diagnosis of heart disease.

Study Design

This will be a qualitative study, which will use both focus group and interview designs. The topic guides were developed based on the study objectives, with an even split between (1) patient/clinician experiences of diagnosis and (2) perspectives on the use of digital technologies within healthcare.

Study Population

Based on the available time for data collection against the wider project deadlines, we plan to conduct four focus groups with individuals with lived experience of heart disease. We aim to recruit 5-6 individuals per focus group to ensure there is enough time for each participant to share their views and experiences. The following inclusion/exclusion criteria will be applied: Inclusion criteria: Lifetime diagnosis of heart disease (including but not limited to: angina, heart failure, valve disease, abnormal heart rhythms); aged 18 or over; able to speak English at a level sufficient for participation; able to give informed consent for participation. Exclusion criteria: Major cognitive impairment or dementia preventing participation.

Based on the research team's previous experience of conducting qualitative research with clinicians, a pragmatic decision has been made to aim for a target of 10 clinicians who have had any experience (past or present) of diagnosing people with heart disease to be enrolled into interviews. The eligibility criteria for inclusion are at least 6 months of experience in working with heart disease patients in any capacity (including cardiology, nursing, primary care, or other healthcare professional in multidisciplinary teams); aged 18 or over; able to speak fluent English; and able to give informed consent for participation.

For peer review only

Procedure

As we are interested in hearing from participants across a range of disease durations (i.e., those with very early symptoms and those who are on established management plans), we plan to recruit via: social media platforms such as LinkedIn, Twitter and Instagram; Prolific; and existing cohorts of people from the investigators' previous research studies who have consented to be contacted for research purposes. Clinicians will be recruited using purposive sampling via personal and professional connections, and social media platforms such as LinkedIn, Twitter and Instagram.

All focus groups and interviews will be carried out online over Zoom, and consent and baseline demographic data will also be collected via online Qualtrics surveys prior to the online study. We expect focus groups to take about two hours and interviews to take about one hour. The focus groups and interviews will be semi-structured and will follow a pre-approved question schedule, split into two sections – patient experience of heart disease and views on digital technologies within healthcare (see Appendix). The same researcher will facilitate all the sessions.

Recruitment and data collection began after ethical approval on 14/11/2022 and will continue until the end of March 2023.

Data Analysis Plan

Descriptive statistics for demographics, current mental distress levels, confidence using technologies, and participant type-specific questions (including length of time in clinical role for clinicians and details on health condition for participants with lived-experience of heart disease) will be presented.

Transcriptions from both focus group and interview recordings will be validated by the team of researchers and coded and analysed using the NVivo software. In line with Braun and Clarke's 2006 recommendations (40), an inductive thematic analysis approach will be taken, whereby the data from the transcripts will decide the themes, instead of basing them on any previous theoretical basis.

For peer review only

Ethics and Dissemination

This study was reviewed and approved by the Sciences & Technology Cross-School Research Ethics Council at the University of Sussex (reference ER/FM409/1) on 14/11/2022. We intend to write the resulting paper according to the Consolidated Criteria for Reporting Qualitative research (COREQ) guidelines (41).

Acknowledgements:

We would like to thank the two patient and public involvement groups that helped to inform the design of this study: The NIHR Maudsley Biomedical Research Centre's Race, Ethnicity and Diversity (READ) advisory group and the Sheffield-based Cardiology Patient group.

Author Statement:

Conceptualisation: F Matcham, TJ Chico, J Condell, O Buckley, V Diaz, R Van Arkel

Methodology: F Matcham, TJ Chico

Investigation: K Abdullayev, M Manktelow

Writing – original draft: K Abdullayev

Writing – review and editing: F Matcham, T.J Chico

Supervision: F Matcham, TJ Chico

Project Administration: K Abdullayev, F Matcham

Funding Acqusition: F Matcham, TJ Chico, J Condell, O Buckley, V Diaz, R Van Arkel

Funding Statement:

This work is supported by the UK Engineering and Physical Sciences Research Council (ESPRC) (grant number: EP/X000257/1).

Competing Interests Statement:

There are no conflicts of interest

For peer review only - http://bmjopen.bmj.com/site/about/guidelines.xhtml

Word Count:

1628

References

1. World Health Organisation (WHO). Cardiovascular diseases (CVDs) [Internet]. 2011 [cited 2023 Feb 3]. Available from: https://www.who.int/news-room/fact-sheets/detail/cardiovascular-diseases-(cvds)
2. Health Intelligence Team B. Our vision is a world free from the fear of heart and circulatory diseases. UK Factsheet. 2022.
3. Stafford M, Soljak M, Pledge V, Mindell J. Socio-economic differences in the health-related quality of life impact of cardiovascular conditions. Eur J Public Health [Internet]. 2012 Jun 1 [cited 2023 Feb 3];22(3):301–5. Available from: https://academic.oup.com/eurpub/article/22/3/301/505231
4. Pandya A, Gaziano TA, Weinstein MC, Cutler D. More americans living longer with cardiovascular disease will increase costs while lowering quality of life. Health Aff. 2013 Aug 2;32(10):1706–14.
5. Tušek-Bunc K, Petek D. Comorbidities and characteristics of coronary heart disease patients: Their impact on health-related quality of life. Health Qual Life Outcomes. 2016;14(1).
6. Annapureddy A, Valero-Elizondo J, Khera R, Grandhi GR, Spatz ES, Dreyer RP, et al. Association Between Financial Burden, Quality of Life, and Mental Health Among Those With Atherosclerotic Cardiovascular Disease in the United States. Circ Cardiovasc Qual Outcomes [Internet]. 2018 Nov 1 [cited 2023 Feb 3];11(11):e005180. Available from: https://www.ahajournals.org/doi/abs/10.1161/CIRCOUTCOMES.118.005180
7. Li C, Young BR, Jian W. Association of socioeconomic status with financial burden of disease among elderly patients with cardiovascular disease: Evidence from the China Health and Retirement Longitudinal Survey. BMJ Open [Internet]. 2018 [cited 2023 Feb 3];8(3):18703. Available from: http://bmjopen.bmj.com/
8. Huffman MD, Rao KD, Pichon-Riviere A, Zhao D, Harikrishnan S, Ramaiya K, et al. A

For peer review only

cross-sectional study of the microeconomic impact of cardiovascular disease hospitalization in four low- and middle-income countries. PLoS One [Internet]. 2011 [cited 2023 Feb 3];6(6):e20821. Available from: https://journals.plos.org/plosone/article?id=10.1371/journal.pone.0020821

9. Mszar R, Grandhi GR, Valero-Elizondo J, Caraballo C, Khera R, Desai N, et al. Cumulative burden of financial hardship from medical bills across the spectrum of diabetes mellitus and atherosclerotic cardiovascular disease among non-elderly adults in the United States. J Am Heart Assoc [Internet]. 2020 May 18 [cited 2023 Feb 3];9(10):15523. Available from: https://www.ahajournals.org/doi/abs/10.1161/JAHA.119.015523

10. Miller J, Edwards LD, Agustí A, Bakke P, Calverley PMA, Celli B, et al. Comorbidity, systemic inflammation and outcomes in the ECLIPSE cohort. Respir Med. 2013 Sep 1;107(9):1376–84.

11. Haffner SM. Relationship of metabolic risk factors and development of cardiovascular disease and diabetes. [Internet]. Vol. 14 Suppl 3, Obesity (Silver Spring, Md.). John Wiley & Sons, Ltd; 2006 [cited 2023 Feb 3]. p. 121S-127S. Available from: https://onlinelibrary.wiley.com/doi/full/10.1038/oby.2006.291

12. Buddeke J, Bots ML, van Dis I, Liem A, Visseren FLJ, Vaartjes I. Trends in comorbidity in patients hospitalised for cardiovascular disease. Int J Cardiol. 2017 Dec 1;248:382–8.

13. De Hert M, Detraux J, Vancampfort D. The intriguing relationship between coronary heart disease and mental disorders. Dialogues Clin Neurosci [Internet]. 2018 Mar 1 [cited 2023 Feb 3];20(1):31–40. Available from: https://www.tandfonline.com/doi/abs/10.31887/DCNS.2018.20.1/mdehert

14. Hansen S. Mental health issues associated with cardiovascular disease in women [Internet]. Vol. 26, Psychiatric Clinics of North America. Elsevier; 2003 [cited 2023 Feb 3]. p. 693–712. Available from: http://www.psych.theclinics.com/article/S0193953X03000376/fulltext

15. Huang KL, Su TP, Chen TJ, Chou YH, Bai YM. Comorbidity of cardiovascular diseases with mood and anxiety disorder: A population based 4-year study. Psychiatry Clin Neurosci [Internet]. 2009 Jun 1 [cited 2023 Feb 3];63(3):401–9. Available from: https://onlinelibrary.wiley.com/doi/full/10.1111/j.1440-1819.2009.01974.x

For peer review only

16. Möller-Leimkühler AM. Gender differences in cardiovascular disease and comorbid depression. Dialogues Clin Neurosci [Internet]. 2007 [cited 2023 Feb 3];9(1):71–83. Available from: https://www.tandfonline.com/doi/abs/10.31887/DCNS.2007.9.1/ammoeller

17. McGill HC, McMahan CA, Gidding SS. Preventing heart disease in the 21st century: Implications of the pathobiological determinants of atherosclerosis in youth (PDAY) study [Internet]. Vol. 117, Circulation. Lippincott Williams & Wilkins; 2008 [cited 2023 Feb 3]. p. 1216–27. Available from: https://www.ahajournals.org/doi/abs/10.1161/CIRCULATIONAHA.107.717033

18. Karunathilake SP, Ganegoda GU. Secondary Prevention of Cardiovascular Diseases and Application of Technology for Early Diagnosis. Martinez E, editor. Biomed Res Int [Internet]. 2018;2018:5767864. Available from: https://doi.org/10.1155/2018/5767864

19. Muhammad Y, Almoteri M, Mujlid H, Alharbi A, Alqurashi F, Dutta AK, et al. An ML-Enabled Internet of Things Framework for Early Detection of Heart Disease. Biomed Res Int [Internet]. 2022 [cited 2023 Feb 3];2022:12. Available from: https://doi.org/10.1155/2022/3372296

20. Muhammad Y, Tahir M, Hayat M, Chong KT. Early and accurate detection and diagnosis of heart disease using intelligent computational model. Sci Rep [Internet]. 2020 [cited 2023 Feb 3];10(1). Available from: https://doi.org/10.1038/s41598-020-76635-9

21. Mensah GA, Wei GS, Sorlie PD, Fine LJ, Rosenberg Y, Kaufmann PG, et al. Decline in Cardiovascular Mortality: Possible Causes and Implications [Internet]. Vol. 120, Circulation Research. Lippincott Williams & Wilkins Hagerstown, MD; 2017 [cited 2023 Feb 3]. p. 366–80. Available from: https://www.ahajournals.org/doi/abs/10.1161/CIRCRESAHA.116.309115

22. Ergin A, Muntner P, Sherwin R, He J. Secular trends in cardiovascular disease mortality, incidence, and case fatality rates in adults in the United States. Am J Med. 2004 Aug 15;117(4):219–27.

23. Gilthorpe MS, Wilson RC. Rural/urban differences in the association between deprivation and healthcare utilisation. Soc Sci Med. 2003 Dec 1;57(11):2055–63.

24. Farmer J, Iversen L, Campbell NC, Guest C, Chesson R, Deans G, et al. Rural/urban

For peer review only

differences in accounts of patients' initial decisions to consult primary care. Heal Place. 2006 Jun 1;12(2):210–21.

25. Kaufman EA, McDonell MG, Cristofalo MA, Ries RK. Exploring barriers to primary care for patients with severe mental illness: Frontline patient and provider accounts. Issues Ment Health Nurs [Internet]. 2012 Mar [cited 2023 Feb 3];33(3):172–80. Available from: https://www.tandfonline.com/doi/abs/10.3109/01612840.2011.638415

26. Miller CL, Druss BG, Dombrowski EA, Rosenheck RA. Barriers to primary medical care among patients at a community mental health center. Psychiatr Serv [Internet]. 2003 Aug 1 [cited 2023 Feb 3];54(8):1158–60. Available from: https://ps.psychiatryonline.org/doi/10.1176/appi.ps.54.8.1158

27. Ross LE, Vigod S, Wishart J, Waese M, Spence JD, Oliver J, et al. Barriers and facilitators to primary care for people with mental health and/or substance use issues: A qualitative study. BMC Fam Pract. 2015;16(1).

28. Klersy C, De Silvestri A, Gabutti G, Regoli F, Auricchio A. A Meta-Analysis of Remote Monitoring of Heart Failure Patients. J Am Coll Cardiol. 2009 Oct 27;54(18):1683–94.

29. Kulshreshtha A, Kvedar JC, Goyal A, Halpern EF, Watson AJ. Use of remote monitoring to improve outcomes in patients with heart failure: A pilot trial. Int J Telemed Appl [Internet]. 2010 Jan 1 [cited 2023 Feb 3];2010. Available from: https://dl.acm.org/doi/10.1155/2010/870959

30. Best J. Wearable technology: Covid-19 and the rise of remote clinical monitoring [Internet]. Vol. 372, The BMJ. British Medical Journal Publishing Group; 2021 [cited 2023 Feb 3]. Available from: https://www.bmj.com/content/372/bmj.n413

31. Chidambaram S, Erridge S, Kinross J, Purkayastha S. Observational study of UK mobile health apps for COVID-19 [Internet]. Vol. 2, The Lancet Digital Health. Elsevier Ltd; 2020 [cited 2023 Feb 3]. p. e388–90. Available from: http://www.thelancet.com/article/S2589750020301448/fulltext

32. Powell J, Newhouse N, Boylan A-M, Williams V. Digital health citizens and the future of the NHS. Digit Heal [Internet]. 2016 [cited 2023 Feb 3];2:205520761667203. Available from: https://us.sagepub.com/en-us/nam/open-access-at-sage

33. Islam SMS, Maddison R. Digital health approaches for cardiovascular diseases prevention and management: Lessons from preliminary studies [Internet]. Vol. 7,

For peer review only

mHealth. AME Publications; 2021 [cited 2023 Feb 3]. Available from: /pmc/articles/PMC8326947/

34. Zwack CC, Haghani M, Hollings M, Zhang L, Gauci S, Gallagher R, et al. The evolution of digital health technologies in cardiovascular disease research [Internet]. Vol. 6, npj Digital Medicine. Nature Publishing Group; 2023 [cited 2023 Feb 3]. p. 1–11. Available from: https://www.nature.com/articles/s41746-022-00734-2

35. Whitelaw S, Pellegrini DM, Mamas MA, Cowie M, Van Spall HGC. Barriers and facilitators of the uptake of digital health technology in cardiovascular care: a systematic scoping review. Eur Hear J - Digit Heal [Internet]. 2021 May 4 [cited 2023 Feb 3];2(1):62–74. Available from: https://academic.oup.com/ehjdh/article/2/1/62/6128570

36. Bhavnani SP. Digital Health: Opportunities and Challenges to Develop the Next-Generation Technology-Enabled Models of Cardiovascular Care [Internet]. Vol. 16, Methodist DeBakey cardiovascular journal. Houston Methodist DeBakey Heart & Vascular Center; 2020 [cited 2023 Feb 3]. p. 296–303. Available from: https://journal.houstonmethodist.org/articles/10.14797/mdcj-16-4-296

37. Dagher L, Nedunchezhian S, El Hajjar AH, Zhang Y, Deffer O, Russell A, et al. A cardiovascular clinic patients' survey to assess challenges and opportunities of digital health adoption during the COVID-19 pandemic. Cardiovasc Digit Heal J [Internet]. 2022 Feb 1 [cited 2023 Feb 3];3(1):31–9. Available from: http://www.cvdigitalhealthjournal.com/article/S2666693621001249/fulltext

38. Gilbey D, Morgan H, Lin A, Perry Y. Effectiveness, acceptability, and feasibility of digital health interventions for LGBTIQ+ young people: Systematic review [Internet]. Vol. 22, Journal of Medical Internet Research. Journal of Medical Internet Research; 2020 [cited 2023 Feb 3]. p. e20158. Available from: https://www.jmir.org/2020/12/e20158

39. Gentili C, Zetterqvist V, Rickardsson J, Holmström L, Simons LE, Wicksell RK. ACTsmart – development and feasibility of digital Acceptance and Commitment Therapy for adults with chronic pain. npj Digit Med [Internet]. 2020 Feb 13 [cited 2023 Feb 3];3(1):1–12. Available from: https://www.nature.com/articles/s41746-020-0228-4

40. Braun V, Clarke V. Using thematic analysis in psychology. Qualitative research in psychology. 2006 Jan 1;3(2):77-101.

For peer review only - http://bmjopen.bmj.com/site/about/guidelines.xhtml

For peer review only

41. Tong A, Sainsbury P, Craig J. Consolidated criteria for reporting qualitative research (COREQ): a 32-item checklist for interviews and focus groups. International journal for quality in health care. 2007 Dec 1;19(6):349-57.

For peer review only

Digital Twin Question Schedules

Lived Experience Focus Group Questions

Heart Disease-related Questions (diagnosis process)

1. Please describe the series of events which took place that led to your heart disease diagnosis
   - Possible prompts: how involved did you feel in this process, active/passive, taken seriously by clinicians/dismissed, big/small role

2. How did you keep track of your symptoms before you were given your diagnosis?
   - Possible prompts: digital tools used, manual log, own method

3. After you first brought up concerns about your heart to your doctor, what did they ask you to do, if anything?
   - Possible prompts: digital tools used, manual log, own method

4. What types of numeric data did your doctor discuss with you, if any?
   - Possible prompts: percentage risks, heart rates, blood pressure, etc
   - Follow up: Was the meaning/importance of the data clear to you?

5. What other aspects of your overall health and wellbeing were important to you around the time you were getting diagnosed?
   - Possible prompts: existing comorbidities, mental health, social support, stress, financial status, lifestyle choices, health behaviours etc

6. During the diagnosis process, what were some important things/people that made the experience less difficult?
   - Possible prompts: staff, communication, social support at home

7. What were the greatest challenges for you during the diagnosis process?
   - Possible prompts: communication, access to information/emotional support, dealing with clinicians, difficulty understanding medical terminology

8. What were your experiences with doctors, nurses and other healthcare staff during the process of getting diagnosed?
   - Possible prompts: positive/negative experience, specific experiences standing out, communication, prejudices or discrimination?
   - Follow up (depending on previous responses) – how did this impact the rest of your treatment?
   - Follow up (depending on previous responses) – how has communication with your clinician impacted your treatment?

9. What do you feel delays or prevents people from getting accurate diagnoses?
   - Possible prompts: access to healthcare, language barriers, fear of stigma/discrimination, lack of concern for health, financial burdens, lack of social support

For peer review only

Digital Twin Question Schedules

10. How do you think we could address these things to help someone get a quicker and more accurate diagnosis?
    - Possible prompts: better, more objective data, easier to explain problems to clinicians, access to healthcare, language assistant, addressing stigmas, financial support, social/mental health/emotional support

Digital Technologies Questions

1. Technologies such as televisions, computers, smartphones etc. have become increasingly integral to our daily lives.
   How do you feel about using different types of technologies in your life?
   - Possible prompts: positive/negative, certain elements easier/harder

2. More specifically, digital technologies are those that can electronically generate, process and store different types of data. Digital technologies are increasingly being used in the healthcare sector.
   What experiences do you have using digital health tools within your healthcare treatment (doesn't have to be specific to heart disease diagnosis)?
   - Possible prompts: positive/negative, limited use, fit for purpose, implementation by clinicians, clinician experience with them

3. Thinking back to the challenges we mentioned in the first part of our discussions [give examples], do you feel digital technologies would help improve, or make worse, any of these issues? If so, which ones and how?
   - Note down issues mentioned and prompt those if needed

Digital health tools are being used more and more in the healthcare sector, as they can improve the way we diagnose and monitor many conditions (including heart disease) by tracking symptoms more effectively.

4. If you were given a digital health tool to help improve the accuracy of your heart disease diagnosis, what sort of things would put you off from using it?
   - Possible prompts: digital literacy, not seen as valuable, preference to deal with a real person, health status, financial status

5. What would motivate you to use it?
   - Possible prompts: incentives, feedback, avatar

6. How would you feel about getting feedback from this type of technology?
   - Follow up: how would it impact your engagement with it?
   - Possible prompts: agency in diagnosis, better awareness of condition/symptoms, more accurate monitoring

7. How would you feel about getting feedback from this device before seeing your doctor?
   - Possible prompts: examples like not enough movement, try to stand more etc;

For peer review only

Digital Twin Question Schedules

8. Who do you think should receive the information that comes from this device?
   - Prompts: clinician, patient, family

9. Do you think there will be any challenges when trying to include a digital monitoring tool in daily patient use?
   - Follow up: What factors do you think we need to consider to make it as easy to use as possible?

The final two questions will be more specific to the final product we are aiming to create. Try to imagine you have a wearable device that is linked to a smartphone app, which allows you to enter data about your symptoms, how you are feeling, certain stressors that may be occurring in your day and so on. This data would all be processed by the app and then reported back to you and your clinician in an easy-to-read format. Let's discuss what you think about specific elements that might be included…

10. If the data were to be sent to you, what would be the most helpful way for the data to be presented/communicated to you?
    - Possible prompts: features like colours, layout etc., how to communicate it – email summary, app interface etc, would you want to see it yourself before seeing your clinician or not?

11. What are your thoughts on having a virtual doctor/avatar/chatbot to interact with on a device?
    - Follow up: do you think being able to personalise the avatar would help with engagement and relatability of the virtual element?
    - Follow up: do you think this would be better than having a simple logging/tracking system like they use for other health tracking apps? Or more complicated?
    - Follow up: any immediate deterrents come to mind from having a 'chatbot' type function?

For peer review only

Digital Twin Question Schedules

Clinician Interview Questions

Heart Disease-related Questions (diagnosis process)

1. Please describe the series of events that normally take place when you diagnose a patient with heart disease.
    - Possible prompts: factors into your decision, length of time, with collaboration/advice from other colleagues, how it's changed with years of experience.

2. How do you monitor your patients' symptoms prior to/following diagnosis?
    - Follow up: what do you ask them to do?
    - Possible prompts: digital tools used, manual log, own method

3. Which numeric data do you find most valuable when making diagnoses?
    - Possible prompts: ECGs, heart rate, blood pressure etc.

4. What other aspects of overall patient health and wellbeing are important to you when you are making a heart disease diagnosis?
    - Possible prompts: existing comorbidities, mental health, social support, stress, financial status, lifestyle choices, health behaviours etc

5. What do you feel are important factors for ensuring a patient has a positive experience following a heart disease diagnosis?
    - Possible prompts: staff, communication, social support at home

6. What are the greatest challenges for you, as a clinician, during the diagnosis process?
    - Possible prompts: communication, medical advice adherence, language barriers, implicit biases due to potentially 'self-inflicted' nature of disease

7. What are your interactions with your patients like prior to/following their heart disease diagnosis?
    - Possible prompts: positive/negative experience, specific experiences standing out, communication, prejudices or discrimination?
    - Follow up (depending on previous responses) – how did this impact the rest of their treatment?
    - Follow up (depending on previous responses) – how has communication with your patients impacted your treatment?

8. What do you feel are potential barriers that prevent people from getting accurate diagnoses?
    - Possible prompts: access to healthcare, language barriers, fear of stigma/discrimination, lack of concern for health, financial burdens, lack of social support

For peer review only

Digital Twin Question Schedules

9. How do you think we could address these barriers to help someone get an accurate diagnosis faster?
   - Possible prompts: better, more objective data, easier to explain problems to clinicians, access to healthcare, language assistant, addressing stigmas, financial support, social/mental health/emotional support

Digital Technologies Questions

1. Technologies such as televisions, computers, smartphones etc. have become increasingly integral to our daily lives.
   How do you feel about using different types of technologies in your life?
   - Possible prompts: positive/negative, certain elements easier/harder

2. What experiences do you have using digital health tools within your healthcare profession? (doesn't have to be specific to heart disease diagnosis)
   - Possible prompts: positive/negative, limited use, fit for purpose, implementation by clinicians, clinician experience with them

3. Thinking back to the challenges we mentioned in the first part of our discussions [give examples], do you feel digital technologies would help improve, or make worse, any of these issues? If so, which ones and how?
   - Note down issues mentioned and prompt those if needed

As you may know, digital health tools are being used more and more in the healthcare sector, to improve the way we diagnose and monitor many conditions (including heart disease) by tracking symptoms more effectively.

4. What would prevent you from using/prescribing a digital health tool made for improving diagnosis of heart disease?
   - Possible prompts: digital literacy, not seen as valuable, preference to deal with a real person, health status, financial status

5. What do you think would prevent your patients from engaging with a digital health tool for symptom monitoring?
   - Possible prompts: digital literacy, not seen as valuable, preference to deal with a real person, health status, financial status

6. What would motivate you to engage in such technologies?
   - Possible prompts: incentives, feedback, avatar, certain data types/presentation/summaries

7. What do you think would motivate your patients to engage in such technologies?
   - Possible prompts: incentives, feedback, avatar

8. Do you feel that patients getting feedback from a digital monitoring system would improve engagement with it? If so, how, why/why not?
   - Possible prompts: agency in diagnosis, better awareness of condition/symptoms, more accurate monitoring

For peer review only

Digital Twin Question Schedules

9. How would you feel if your patients were getting feedback from the digital monitoring system before seeing you?
    - Possible prompts: examples like not enough movement, try to stand more etc;

10. Do you foresee any challenges with implementing a digital monitoring tool into daily patient use? What factors do you think we need to consider to make it as easy to use as possible?

The final two questions will be more specific to the final product we are aiming to create. Try to imagine you will be giving patients a wearable device that is linked to a smartphone app, which allows them to enter data about their symptoms, how they are feeling, certain stressors that may be occurring in their day and so on. This data would all be processed by the app and then reported back to you and your patient in an easy-to-read format. Let's discuss what you think about specific elements that might be included…

11. What are your thoughts on having a virtual doctor/avatar/chatbot to interact with on a device?
    - Follow up: do you think being able to personalise the avatar would help with engagement and relatability of the virtual element?
    - Follow up: do you think this would be better than having a simple logging/tracking system like they use for other health tracking apps? Or more complicated?
    - Follow up: any immediate deterrents come to mind from having a 'chatbot' type function?

12. What would be the most helpful way for the data to be presented/communicated to you?
    - Follow up: how do you think this should differ from how data is presented to patients, if at all?
    - Possible prompts: features like colours, layout etc., how to communicate it – email summary, app interface etc, would you want to see it yourself before seeing your clinician or not?

For peer review only
